# Vitamin D and Endothelial Function

**DOI:** 10.3390/nu12020575

**Published:** 2020-02-22

**Authors:** Do-Houn Kim, Cesar A. Meza, Holly Clarke, Jeong-Su Kim, Robert C. Hickner

**Affiliations:** 1 Department of Nutrition, Food and Exercise Sciences, Florida State University, Tallahassee, FL 32306, USA; dkim5@fsu.edu (D.-H.K.); cm18dq@my.fsu.edu (C.A.M.); hec17e@my.fsu.edu (H.C.); jkim6@fsu.edu (J.-S.K.); 2 Center for Advancing Exercise and Nutrition Research on Aging, Florida State University, Tallahassee, FL 32306, USA; 3 Institute of Sports Sciences and Medicine, College of Human Sciences, Florida State University, Tallahassee, FL 32306, USA; 4 Department of Biokinetics, Exercise and Leisure Sciences, School of Health Sciences, University of KwaZulu-Natal, Westville 4041, South Africa

**Keywords:** calcitriol, endothelial dysfunction, nitric oxide, NO, NOX, oxidative stress, ROS, inflammation, vitamin D deficiency, eNOS

## Abstract

Vitamin D is known to elicit a vasoprotective effect, while vitamin D deficiency is a risk factor for endothelial dysfunction (ED). ED is characterized by reduced bioavailability of a potent endothelium-dependent vasodilator, nitric oxide (NO), and is an early event in the development of atherosclerosis. In endothelial cells, vitamin D regulates NO synthesis by mediating the activity of the endothelial NO synthase (eNOS). Under pathogenic conditions, the oxidative stress caused by excessive production of reactive oxygen species (ROS) facilitates NO degradation and suppresses NO synthesis, consequently reducing NO bioavailability. Vitamin D, however, counteracts the activity of nicotinamide adenine dinucleotide phosphate (NADPH) oxidase which produces ROS, and improves antioxidant capacity by enhancing the activity of antioxidative enzymes such as superoxide dismutase. In addition to ROS, proinflammatory mediators such as TNF-α and IL-6 are risk factors for ED, restraining NO and eNOS bioactivity and upregulating the expression of various atherosclerotic factors through the NF-κB pathway. These proinflammatory activities are inhibited by vitamin D by suppressing NF-κB signaling and production of proinflammatory cytokines. In this review, we discuss the diverse activities of vitamin D in regulating NO bioavailability and endothelial function.

## 1. Introduction

Vitamin D deficiency has been linked to many metabolic and cardiovascular diseases (CVD), and therefore supplemental vitamin D has been used in the treatment and prevention of these diseases. This fat-soluble vitamin is a steroid hormone that is endogenously produced in the skin of humans. Initially, researchers have focused on a link between low levels of vitamin D and the occurrence of bone disease, as vitamin D has been primarily known for its role in regulating calcium homeostasis and bone metabolism. Data from the 2005–2006 National Health and Nutrition Examination Survey indicate that vitamin D deficiency, defined as a serum 25-hydroxyvitamin D levels ≤ 20 ng/mL (50 nmol/L), is common among adults aged 20 years and over in the United States (US), with 41.6% of adults reporting a vitamin D deficiency [1]. This vitamin D deficiency epidemic can be attributable to factors such as poor sunlight exposure, insufficient intake of vitamin-containing foods and malabsorption syndromes such as Crohn’s disease and celiac disease [2].

Preclinical studies have revealed that vitamin D exerts its physiological effects through the vitamin D receptors (VDR) [3], and these receptors were found not only within bone but also within other types of tissues such as skeletal muscle and vascular endothelial cells [4,5]. Emerging evidence in recent studies investigating extraskeletal actions of vitamin D suggest that insufficient vitamin D levels are linked to the development of CVD [6,7]. Endothelial dysfunction (ED) is a potent risk factor for CVD and characterized by reduced bioavailability of vasodilators, particularly nitric oxide (NO) [8]. ED is known to predispose blood vessels to the development of atherosclerosis and is an important prognostic marker for CVD [9]. In recent years, observational studies have shown a plausible relationship between endothelial function and vitamin D status. Zhang et al. reported that circulating 25-hydroxyvitamin D concentrations were inversely associated with ED assessed by brachial artery flow-mediated dilation (FMD) in individuals with non-dialysis chronic kidney disease [10]. Similar relationships between vitamin D status and endothelial function have been shown in normotensive adult females in which endothelial vasodilator function was measured through reactive hyperemia index (RHI), assessed by pulse arterial tonometry [11]. In addition, endothelial cells with an absence of the endothelial VDR have shown impaired vasodilatory response to perfused acetylcholine (ACh) [5]. Thus, a growing body of research supports the link between vitamin D and endothelial function, and a deficiency in serum vitamin D levels has been associated with CVD. Although there is ample evidence suggesting the link between vitamin D and endothelial function, the exact underlying mechanisms by which vitamin D may affect endothelial function is not yet completely understood. Thus, we review the available literature and present the physiological roles of vitamin D in regulating endothelial function.

## 2. Basic Physiology of Vitamin D and the Endothelium

The majority of vitamin D in the body is obtained through the sunlight-initiated biosynthesis in the skin. When the skin is exposed to the sun, a cholesterol precursor, 7-dehydrocholesterol, is converted to previtamin D_3_ and vitamin D_3_ (cholecalciferol) by ultraviolet B (UVB) radiation and thermal stimulation, respectively [12]. Less than 30% of vitamin D can be obtained through diet [13]. Vitamin D found in foods can exist in two forms: Vitamin D_2_ (ergocalciferol), found in vegetable sources such as sun-dried mushrooms; and vitamin D_3_, found mostly in oil-rich fish. Both vitamin D_2_ and D_3_ go through hydroxylation twice to become the biologically active form, namely 1α,25-dihydroxyvitamin D_3_ (1α,25(OH)_2_D_3_ or calcitriol) [14]. The first phase of hydroxylation occurs in the liver in which vitamin D is converted to calcidiol [25(OH)D] by 25-hydroxylase, and the second phase of hydroxylation, catalyzed by 1α-hydroxylase, occurs mainly in the kidney, which produces 1α,25(OH)_2_D_3_ from 25(OH)D; 1α,25(OH)_2_D_3_ then exerts biological actions by binding to nuclear VDR or plasma membrane VDR. The principal biological actions of 1α,25 (OH)_2_D_3_ are mediated by the nuclear VDR through which 1α,25(OH)_2_D_3_ controls gene expression. The ligand-bound nuclear VDR translocate into the nucleus and form homodimers or heterodimers with the retinoid X receptor (RXR). After this nuclear dimerization, the homodimers or VDR-RXR heterodimers bind to specific enhancer elements found in the promoter region of vitamin D-regulated genes, referred to as vitamin D response elements (VDRE), thus activating the expression of specific target genes [15]. The genomic action of vitamin D is involved in expression of over 200 genes, the functional activities which play essential roles in various physiological processes including homeostatic control of bone metabolism, immune cell growth, and regulation of vascular tone [5,16,17]. Activation of the plasma membrane VDR can elicit an intracellular signal transduction pathway independent of the VDRE [18,19], although most biological actions of calcitriol are attributed to activation of the nuclear VDR given that the nuclear VDR effectively responds to 1α,25 (OH)_2_D_3_ at sub-nanomolar concentrations [20]. Vitamin D has been implicated in various biological activities within the body, and as such, VDR have been found in most tissues and cells [21]. The most well-known action of vitamin D is associated with the homeostasis of calcium and bone mineralization, with vitamin D and VDR primarily functioning to promote calcium absorption in the intestines [22]. Vitamin D is an essential component necessary for the development, growth, and mineralization of bone during the formative years of childhood; however, vitamin D continues to play a crucial role maintaining optimal bone health throughout the lifespan in adults of all ages [23].

**Figure 1:**
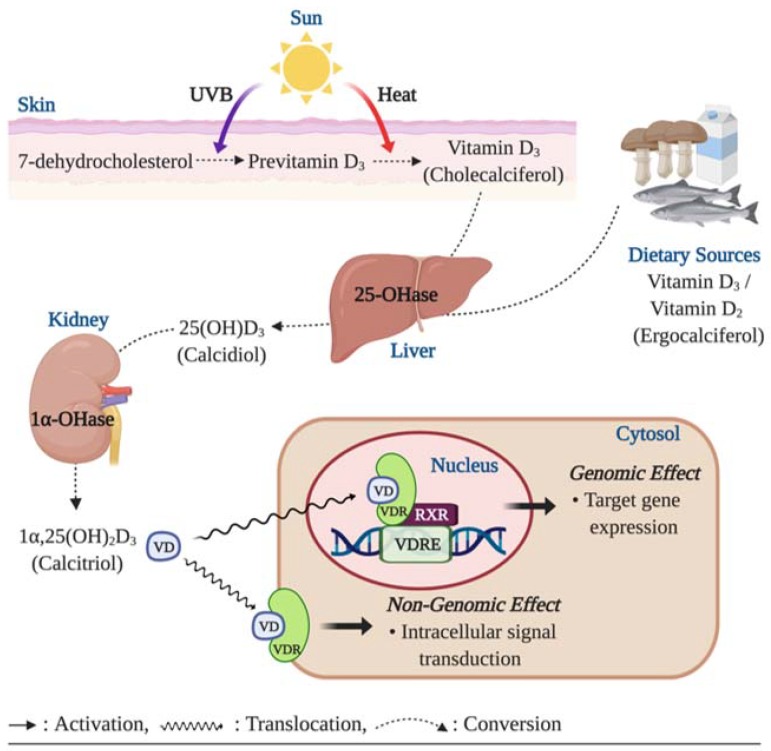
Basic physiology of vitamin D action. Humans obtain vitamin D mainly through endogenous production of previtamin D_3_ and vitamin D_3_ in the skin, followed by subsequent conversions in the liver and kidney. When exposed to the sun, 7-dehydrocholesterol converts to previtamin D_3_ and vitamin D_3_ by ultraviolet B (UVB) radiation and heat, respectively, in the skin. A small amount of vitamin D can be present in natural food as vitamin D_2_ and D_3_. Vitamin D_2_/D_3_ go through hydroxylation two times in the liver (vitamin D_2_/D_3_ → 25 (OH) D_3_) and the kidney (25 (OH) D3 → 1α,25 (OH)_2_D_3_) to become a biologically active form of vitamin D_3_, 1α,25 (OH)_2_D_3_;1α,25 (OH)_2_D_3_ exerts its biological actions by binding to the nuclear vitamin d receptor (VDR), which associates with a retinoid x receptor (RXR) in the nucleus. The VDR/RXR heterodimers bind to the vitamin D response element (VDRE) in the promoter region of vitamin D-regulated genes and initiate expression of various genes. VDR is also found in the plasma membrane, and the liganded plasma membrane VDR activates an intracellular signaling transduction involved in many physiological actions.

Both genomic and non-genomic actions of vitamin D are mediated by the VDR, and ligand-activated VDRs are involved in physiological processes through regulation of transcriptional activity of target genes or activation of intracellular second messengers [24,25]. While vitamin D plays important roles in various cellular functions, the action of vitamin D can be inhibited by a number of factors such as reduced biosynthesis of vitamin D, a lack of vitamin D hydroxylases in the cells, and reduced VDR content; thus, the impaired vitamin D action may contribute to the development of many chronic diseases including osteoporosis, diabetes, atherosclerosis, ED, and cancer [26]. A simplified schematic diagram of the basic physiology of vitamin D is shown in Figure 1. 

The vascular endothelium is composed of a single lining of endothelial cells, standing as a barrier between blood and tissues throughout the entire vascular system. In 1980, Furchgoot and Zawadzki first demonstrated that endothelial cells were essential for vasodilation induced by ACh, a potent endothelium-dependent vasodilator [27]. Since then, the endothelium has been known to play a pivotal role in regulating vascular homeostasis and hemodynamics. A dysfunctional endothelium is strongly linked to the pathogenesis of various CVDs including diabetes, hypertension, atherosclerosis, and peripheral artery diseases [28,29,30]. In the presence of humoral, neural, and mechanical stimuli, the endothelium releases various vasodilatory substances including NO, prostacyclin, C-type natriuretic peptide, and endothelium-derived hyperpolarizing factors (EDHF) as well as vasoconstrictors, such as reactive oxygen species (ROS), angiotensin 2, and endothelin-1 [31,32,33]. A critical balance between these vasodilators and vasoconstrictors is paramount for the regulation of a healthy vascular tone. Any disruption in this balance could lead to impairments in endothelial function, promoting an unhealthy vascular phenotype that could predispose augmented vasoconstriction, formation of plaque, vascular inflammation, and atherosclerosis [33]. Among these substances, NO is a primary vasoactive substance that works as a potent vasodilator in addition to other vasoprotective properties such as protection from vessel inflammation and lesion formation [34]. NO is synthesized from L-arginine by endothelial NO synthase (eNOS) in the endothelium [35]. The endothelium-derived NO acts on adjacent vascular smooth muscle cells in a paracrine manner and induces vascular muscle relaxation through the activation of soluble guanylate cyclase (sGC), which, in turn, leads to an increased production of cyclic guanosine monophosphate (cGMP) and a decrease in intracellular calcium concentrations [36,37,38]. In addition to its potent vasodilatory effect, NO has been proposed to have vasoprotective properties in which NO protects the vessel from developing atherosclerosis by inhibiting platelet adherence and aggregation, and leukocyte activation [39,40,41]. Therefore, reduced bioavailability and bioactivity of NO are primary characteristics of ED. The major contributors of ED include a reduction in the activity of eNOS that results in impaired NO production, as well as oxidative stress caused by excess free radical production that can lead to quenching of NO and reduced NO bioavailability [42].

## 3. Effect of Vitamin D on Regulation of Nitric Oxide (NO) Bioavailability

**Figure 2:**
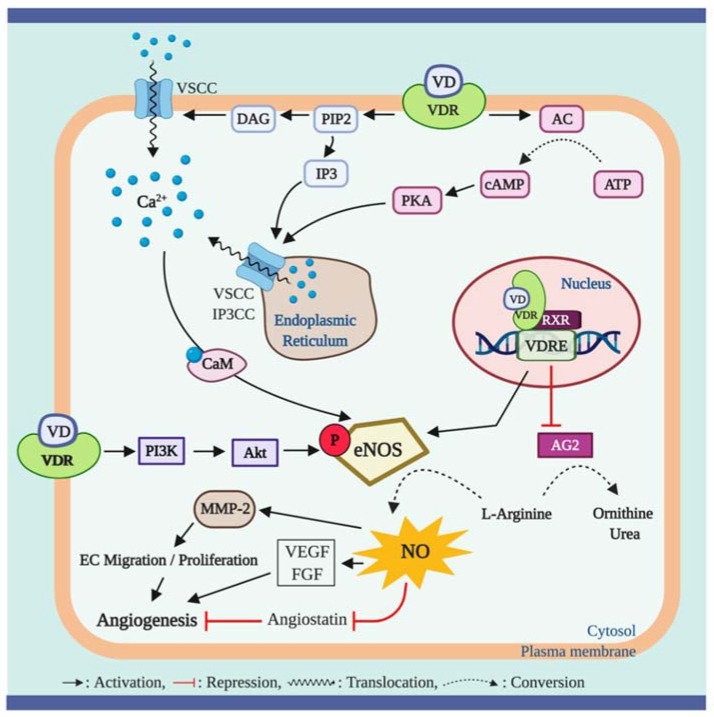
Role of vitamin D and vitamin D receptor (VDR) in regulating nitric oxide (NO) bioavailability. Ligand-bound VDR plays an important role in regulating NO synthesis via alterations in eNOS activity. Activation of plasma membrane VDR upregulates the activity of endothelial NO synthase (eNOS), a calcium dependent enzyme, by upregulating the formation of intracellular second messengers including adenylyl cyclase (AC), diacylglycerol (DAG) and inositol trisphosphate (IP3), which in turn result in calcium influx through the voltage-sensitive calcium channel (VSCC) in the plasma membrane and the sarcoplasmic reticulum and through the ip3 receptor/calcium channel (IP3CC) in the sarcoplasmic reticulum. The increased intracellular calcium concentrations facilitate the calcium-calmodulin (CaM) pathway to activate eNOS. In addition, plasma membrane VDR triggers eNOS activation through phosphorylation of serine-1779 (human serine 1177) on eNOS by activating the phosphoinositide 3-kinase (PI3K)/ protein kinase b (Akt) pathway. Furthermore, genetic action of vitamin D via the nuclear VDR promotes eNOS expression, which synthesizes NO from L-arginine, and suppresses arginase-2 (AG2) expression, which inhibits eNOS activity by hydrolyzing the substrate for NO synthesis (arginine) to ornithine and urea. Increased NO production promotes angiogenesis by upregulating gene expression of matrix metalloproteinase 2 (MMP-2) which improves endothelial cell (EC) migration and proliferation capacity. In addition, NO mediates angiogenetic activity of the cell via upregulation of vascular endothelial growth factor (VEGF) and fibroblast growth factor (FGF) and suppression of the angiogenesis inhibitor, angiostatin.

The role of vitamin D in regulating eNOS dependent NO synthesis is shown in Figure 2. The VDRs, essential for vitamin D action, are expressed in virtually all tissues including endothelial cells, vascular smooth muscle cells, and cardiomyocytes [43]. The ligand-bound VDR works as a transcriptional factor, regulating the expression of specific genes, via binding to the VDRE in the promotor region of target genes [3]. It has been suggested that vitamin D and VDRs could play a key role in regulating NO synthesis via eNOS bioactivity [5,44].

In a pre-clinical study, mice with the absence of endothelial VDR expression displayed reduced bioavailability of NO due to impaired expression of the NO synthesizing enzyme, eNOS, within the endothelium (2). Similarly, when the endothelial-specific VDR was knocked out, reduction in eNOS expression and impaired ACh-induced vasorelaxation were observed in the aorta of mice [5]. The impaired eNOS production capability was recovered by vitamin D treatment in a few laboratory experiments. Martínez-Miguel et al. reported that endothelial cells treated with 10 nM of 1α,25 (OH)_2_D_3_ significantly increased NO production as well as protein abundance and bioactivity of eNOS. Furthermore, this upregulation of NO and eNOS by 1α,25 (OH)_2_D_3_ treatment was directly dependent upon VDR activation, as effects were not detected in the absence of the endothelial VDR [45]. These findings were subsequently tested in an in vivo experiment in which normal Wistar rats treated with 400 ng/kg 1α,25(OH)_2_D_3_, via intraperitoneal injection, showed the similar upregulation of NO and eNOS protein contents in aorta tissues [45]. In addition, the absence of VDR gene has been shown to result in decreased L-arginine bioavailability due to increased arginase-2 expression. This catabolic enzyme is known to compete with eNOS for L-arginine and hydrolyze the substrate to ornithine and urea [44].

In addition to the genomic action of vitamin D, non-genomic actions of vitamin D through the membrane VDR may upregulate intracellular eNOS activity via the intracellular calcium-dependent pathway. The eNOS is classified as a calcium-dependent enzyme [46], and it has been shown that an increased intracellular calcium concentration is critical for increase in NO biosynthesis and induction of vasodilation via eNOS [47]. It has been demonstrated in previous in vitro studies that 1α,25 (OH)_2_D_3_ administration increased intracellular calcium content through the formation of intracellular second messengers, such as cyclic adenosine monophosphate (cAMP), diacylglycerol (DAG), and inositol trisphosphate (IP3) [48]. These signaling molecules are potent activators of protein kinase A (PKA) and protein kinase C (PKC), which trigger calcium release from intracellular stores and calcium uptake through the voltage-sensitive calcium channels in the plasma membrane [49,50]. An increase in the intracellular calcium concentration promotes the formation of calcium/calmodulin (CaM) complex which plays an essential role to activate eNOS [51]. Furthermore, vitamin D may activate eNOS activity in a phosphatidylinositol 3-kinase (PI3K)/protein kinase B (Akt)-dependent fashion. It has been demonstrated that 1α,25 (OH)_2_D_3_ treatment results in activation of the PI3K/Akt pathway in skeletal muscle cells [52,53]. The PI3K/Akt pathway has been shown to induce phosphorylation of serine-1177 to upregulate eNOS activity [54].

Vitamin D has been shown to induce endothelial migration and proliferation, thereby potentially promoting angiogenesis [55]: Vitamin D-induced NO production is, in part, accountable for this physiological process [56,57]. Pro-angiogenic actions of NO have been demonstrated in in vitro and ex vivo experiments which showed that NO prompts the formation of capillary-like structures in human umbilical endothelial cells (HUVEC) and human coronary artery in a 3D matrix [58,59,60]. Potential mechanism underlying the role of NO in angiogenesis involves NO-mediated endothelial cell migration and proliferation [61]. It is worth noting that vitamin D has shown to improve the endothelial cell migration and proliferation by upregulating gene expression of matrix metalloproteinase 2 (MMP-2), an extracellular matrix dissolving factor [56,57]. Furthermore, the increased MMP-2 expression was attributable to increase in NO production in HUVEC and porcine aortic endothelial cells, as the endothelial cell migration and proliferation were abolished by eNOS inhibition with n(ω)-nitro-L-arginine methyl ester (L-NAME) [57,62]. This finding suggests that vitamin D-induced angiogenic activities of endothelial cells are mediated by eNOS-dependent NO production. In addition, NO-mediated formation of new blood vessels involves upregulation of gene expression of angiogenic growth factors including vascular endothelial growth factor (VEGF) and fibroblast growth factor (FGF) and suppression of angiostatin, an endogenous antagonist of angiogenesis [63,64,65].

Transcriptional activity of vitamin D is seen to be effective in increasing eNOS gene expression, thereby upregulating NO production. The upregulated NO production may not only improve endothelial function but may also promote endothelial cell angiogenic activities. The VDR is a key mediator of this process. Although there are currently no data supporting a direct link between non-genomic action of vitamin D and the bioactivity of NO and eNOS, the potential mechanisms of the non-genomic effect of vitamin D on eNOS activity and NO production may be elucidated by investigations into the role of vitamin D in regulating intracellular calcium contents and the eNOS activating signaling pathway.

## 4. Anti-Oxidant Capacity of Vitamin D Reduces Oxidative Stress-Induced Endothelial Dysfunction

**Figure 3:**
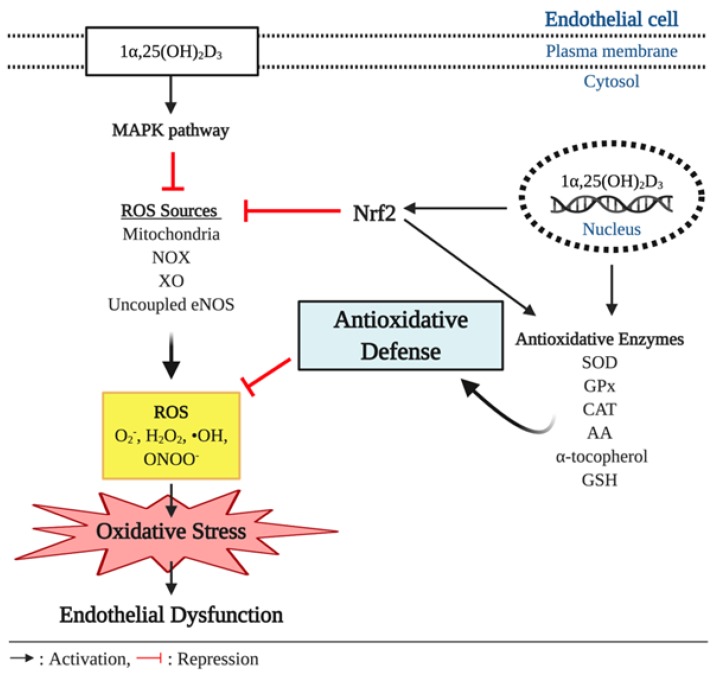
Antioxidant effect of vitamin D and endothelial function. In pathophysiological conditions, overproduction of reactive oxygen species (ROS), such as superoxide anion (O_2_^−^), hydrogen peroxide (H_2_O_2_), hydroxyl radical (•OH), and peroxynitrite (ONOO^−^), outbalances antioxidative defenses and causes oxidative stress, which is implicated to development of endothelial dysfunction. In the cell, ROS are produced by various intracellular sources including mitochondria, nicotinamide adenine dinucleotide phosphate (NADPH) oxidase (NOX), xanthine oxidase (XO), and uncoupled endothelial nitric oxide synthase (eNOS). Vitamin D elicits antioxidant effects through upregulating expression of antioxidative enzymes including superoxide dismutase (SOD), glutathione peroxidase (GPx), catalase (CAT), ascorbic acid (AA), α-tocopherol, and glutathione (GSH) that can scavenge the free radicals. In addition, the genetic action of vitamin D triggers the expression of nuclear respiratory factor 2 (Nrf2), a key transcriptional factor that suppresses ROS production from its various sources and upregulates the expression of the antioxidative enzymes.

Oxidative stress is reflected by a disturbance in the balance between the production of ROS and antioxidative defenses. The overproduction of ROS coupled with inadequate scavenging by antioxidants can adversely affect several biological processes, such as signal transduction, through the oxidation of proteins, lipids, and DNA [66]. The various layers of the vasculature, including the endothelium, smooth muscle cells, and adventitia, are major sources of ROS production, with the predominant intracellular sources of ROS being the mitochondrial respiratory chain and the ROS producing enzymes: Uncoupled NOS, NAPDH oxidases, and xanthine oxidase [67,68,69]. The various forms of ROS include the superoxide anion radical (O_2_^−^), hydrogen peroxide (H_2_O_2_), and hydroxyl radical (•OH). NO and peroxynitrite (ONOO^−^) are considered reactive nitrogen species (RNS) [70]. Under normal physiological conditions, ROS are produced as products of metabolism and therefore play a significant role in mediating signal transduction and homeostasis. In the vasculature, ROS can regulate vascular smooth muscle cell (VSMC) contraction and relaxation as well as VSMC formation via VEGF [71,72,73]. In contrast, under pathological conditions, such as obesity and diabetes, increased ROS production outbalances the antioxidative defense and detrimentally influences cells leading to the pathogenesis of numerous pathological conditions including ED, insulin resistance, atherosclerosis, congestive heart failure, cancer, cardiomyopathy, and ischemic heart disease [74,75,76]. As ROS can negatively influence many physiological systems, it is not surprising that there are defense systems in place to protect from ROS-induced damage. Among the various antioxidants that can scavenge free radicals and thereby regulate the cellular oxidative environment are superoxide dismutase (SOD), glutathione peroxidase (GPx), and catalase (CAT), ascorbic acid (AA) and α-tocopherol, and reduced glutathione (GSH) [77] (Figure 3).

Reduced NO bioavailability is a major indicator of impaired endothelial function and is often the result of heightened oxidative stress. In the endothelium, O_2_^−^ rapidly reacts with NO to generate ONOO^−^, which may cause oxidative damage to proteins, lipids, and DNA [78]. It is noteworthy that the O_2_^−^-NO reaction determines the bioavailability of NO as the biomolecular reaction between O_2_^−^ and NO is three to four times faster than the dismutation of O_2_^−^ by the antioxidant, SOD [79]. Furthermore, ONOO^−^ has been shown to induce the conformational disruption of the eNOS protein and consequently results in uncoupled eNOS that further decreases NO production and exacerbates the increased O_2_^−^ production [80]. Moreover, O_2_^−^ can contribute to the reduced NO synthesis by uncoupling eNOS, reducing eNOS protein expression, upregulating the formation of the eNOS inhibitor, asymmetric dimethylarginine (ADMA), and decreasing the bioavailability of the NOS cofactor, 6R-tetrahydrobiopterin (BH4) [81]. Based on the current findings in experimental studies, O_2_^−^ and ONOO^−^ seem to be predominant free radicals that mediate oxidative stress-induced suppression of NO availability and eNOS activity.

Observational studies have indicated that there is an association between insufficient vitamin D levels and increased oxidative stress or reduced antioxidant capacity [82,83]. A few in vivo and in vitro experiments have indicated biochemical links between the genetic effect of vitamin D and oxidative stress. In a study using diabetic rats, vitamin D injection three times per week for 10 weeks at a dose of 0.2 µg/kg resulted in reduced expression of the ROS synthesizing enzyme NAPDH oxidase in the aorta [84]. The inhibitory effect of vitamin D on NADPH oxidase expression has been reported in the hepatocytes of diabetic mice treated with vitamin D injection for 2.5 months at a dose of 800 IU/kg [85]. In addition, Polidoro et al. reported that vitamin D treatment protected HUVEC from H_2_O_2_-induced oxidative stress and associated apoptosis by inhibiting O_2_^−^ generation through the mitogen-activated protein kinases (MAPK) pathway [86]. Additionally, Kanikarla-Marie et al. have also demonstrated that 1α,25 (OH)_2_D_3_ treatment ameliorated oxidative stress by improving antioxidant capacity in HUVEC under hyperketonemia [87]. In this experiment, HUVEC displayed a significant increase in ROS production when exposed to high concentration of ketone bodies. However, 1α,25 (OH)_2_D_3_ treatment suppressed the ROS over-production in a dose dependent manner, which was attributable to enhanced synthesis of the antioxidant enzyme GSH. The 1α,25 (OH)_2_D_3_ treated HUVEC showed upregulation of glutamate cysteine ligase (GCL) gene expression, a key regulatory enzyme for GSH synthesis. When the GCL catalytic subunit (GCLC) was knocked down by GCLC specific siRNA, the increase in GSH and the suppressed ROS production were dampened. Furthermore, this antioxidative action of 1α,25(OH)_2_D_3_ was blunted in the absence of VDR [87]. Similarly, the positive association between plasma vitamin D concentrations and GSH bioavailability were observed in humans with type 2 diabetes (T2D) [88]. In addition, vitamin D improved antioxidative capacity through the upregulation of nuclear factor erythroid 2-related factor 2 (Nrf2) in diabetic mice [89], a major antioxidant system that regulates the expression of various ROS detoxifying and antioxidant enzymes including NF-kB, hemeoxygenase-1, ubiquitin/PKC-ζ-interacting protein A170, peroxiredoxin 1, the heavy and light chain of ferritin, catalase, and thioredoxin [90,91,92,93,94,95] (Figure 3). The antioxidative potential of vitamin D has been proposed in avascular cells. For instance, vitamin D treatment upregulated antioxidant production and inhibited ROS production in the retinal pigment epithelium (RPE) [96]. In a cell culture study by Tohari et al. the RPE treated with H_2_O_2_ displayed significant increases in ROS activity and cell death and reduction of gene expression of antioxidants including SOD, CAT, and GPx. However, 1α,25 (OH)_2_D_3_ treatment eliminated the detrimental effect of H_2_O_2_-induced oxidative stress on the antioxidant synthesis and ROS formation in the RPE [96] (Figure 3). In addition, 1α,25 (OH)_2_D_3_ treatment has been shown to ameliorate the increased accumulation of ONOO^−^ in neural cells cultured under diabetic conditions [97].

Reduced vitamin D action in the body is linked to increased ROS production and weakened antioxidative capacity, which may cause ED via inhibited eNOS and NO synthesis. This ROS-induced damage to NO and eNOS synthesis has been shown to be reduced with vitamin D treatment through upregulation of antioxidants and downregulation of ROS production. However, some of these benefits of vitamin D treatment need to be examined in the endothelium.

## 5. Anti-Inflammatory Effects of Vitamin D and Endothelial Function

Sustained inflammation for prolonged periods of time (from months to years), named chronic inflammation, is known to cause tissue damage and is associated with the pathogenesis of atherosclerosis, ED, and CVD [98]. The chronic inflammation process is mediated by various factors including cell-derived proinflammatory cytokines such as tumor necrosis factor (TNF)-α, interleukin (IL)-1 and IL-6 [99].

Dysfunctional endothelium has been observed in patients with chronic inflammatory diseases such as psoriasis and rheumatoid arthritis [100]. A systematic review of 20 studies of ED in psoriasis has shown that endothelial function, measured by FMD, was significantly impaired in patients with psoriasis in the majority of studies [101]. Similarly, Bergholm et al. found that patients with rheumatoid arthritis displayed ED, measured by forearm vasodilatory responses to intra-arterial infusion of ACh. After treatment with anti-inflammatory drug such as prednisone, the impaired vasodilatory response was ameliorated along with reduced inflammatory markers, such as TNF-α [102].

**Figure 4:**
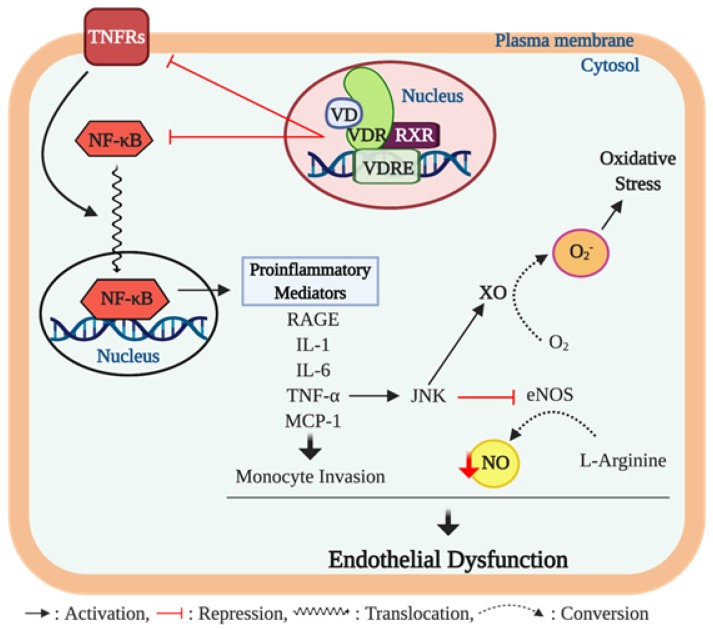
Vitamin D, inflammation, and endothelial dysfunction. The chronic inflammation process contributes to developing endothelial dysfunction through proinflammatory cytokine activity. Ligand bound vitamin D receptor (VDR) activation suppresses gene expression of nuclear factor-κB (NF-κB) and tumor necrosis factor (TNF)-α receptors 2 and 4 (TNFRs). The NF-κB is a key transcription factor that promotes expression of various proinflammatory mediators including advanced glycation end products (AGEs), interleukin (IL)-1 and 6, TNF-α, and monocyte chemoattractant protein-(MCP)-1 which are implicated in endothelial dysfunction. Among these proinflammatory cytokines, TNF-α activates the c-Jun N-terminal kinase (JNK) pathway that inhibits endothelial NO synthase (eNOS) activity, consequently resulting in reduction of nitric oxide (NO) bioavailability. In addition, the upregulated JNK pathway induces the formation of superoxide anion O2− from oxygen (O2) through upregulating xanthine oxidase (XO) activity, which further impairs endothelial function via inducing oxidative stress in the cell. Furthermore, TNF-α bound TNFRs trigger the translocation of NF-κB to the nucleus to promote the expression of proinflammatory mediators.

Kristen et al. have reported that lower serum 25 (OH) D concentrations were associated with ED, assessed by FMD, in healthy middle-aged/older adults; this was, in part, mediated by upregulation of proinflammatory transcription factor NF-κB pathway and the expression of proinflammatory cytokine, IL-6 [103]. Furthermore, the impaired brachial artery FMD was recovered by treatment with a NF-κB inhibitor (i.e., salsalate) to a greater extent in subjects with lower serum 25 (OH) D levels [104]. The NF-κB pathway has been proposed to play a critical role in regulating the proinflammatory-mediated ED [104,105,106] (Figure 4). NF-κB is an important transcriptional factor involved in regulatory gene expression which is responsible for cell adhesion, proliferation, inflammation, and redox status [107]. Activation of the NF-κB pathway results in translocation of the NF-κB heterodimer to the nucleus where it promotes the expression of proinflammatory molecules such as IL-6, TNF-α, monocyte chemoattractant protein 1 (MCP-1), and receptor for advanced glycation end products (AGE) [108,109]. TNF-α has been shown to impair endothelium-dependent NO-mediated vasodilation through the activation of the c-Jun N-terminal kinase (JNK) pathway which inhibits eNOS activation and upregulates O_2_^−^ production via xanthine oxidase in endothelial cells. Furthermore, TNF-α is known to further activate the NF-κB activity, consequently triggering other inflammatory cytokines [110]. In addition, MCP-1 is known to act as a signaling mediator during the progression of TNF-α-mediated ED [111], but also independently exerts detrimental effects on endothelial function by inducing the invasion of monocytes into the endothelial cells [112] (Figure 4).

Emerging evidence indicates that vitamin D may play an important role in modulating inflammation [113,114], and an adequate vitamin D level in the blood is crucial for maintaining the optimal anti-inflammatory response in humans [115]. A few in vitro studies have suggested the potential cellular mechanisms underlying the anti-inflammatory actions of vitamin D in endothelial cells. In cell culture studies, 1α,25 (OH)_2_D_3_ exerts anti-inflammatory effects through suppressed activation of the NF-κB pathway and TNF-α activity and the release of IL-6 in human endothelial cells [116,117]. Similar biochemical actions have been reported in inflammatory cells. In murine macrophages, 1α,25 (OH)_2_D_3_ treatment suppressed the NF-κB translocation to the nucleus resulting in reduced expression of TNF-α [118]. Furthermore, human monocytes treated with 1α,25 (OH)_2_D_3_ suppressed the detrimental effects of TNF-α by inhibiting the expression of its receptors, TNF receptor (TLR)-2 and TLR-4, indicating a potential reduction of proinflammatory cytokine expression from monocytes [119]. Similar results have been reported in human studies, in that monocytes from type 2 diabetic (T2D) patients displayed significantly higher levels of pro-inflammatory cytokines including TNF-α, IL-6, and IL-1 compared to monocytes from a healthy control group; however, treatment with 1α,25 (OH)_2_D_3_ suppressed the expression of the proinflammatory cytokines [120]. Results of an in vivo animal study carried out in diabetic rats demonstrated that hepatic expression of pro-inflammatory mediators such as NF-κB and MCP-1 were significantly higher in diabetic rats compared to healthy controls; however, vitamin D treatment in diabetic rats significantly lowered hepatic expression of the pro-inflammatory mediators [121].

Various chronic inflammatory diseases feature ED, and the activation of the NF-κB pathway as well as proinflammatory cytokines, such as TNF-α, IL-6, and IL-1, have been shown to exert detrimental influences on endothelial function. Anti-inflammatory properties of vitamin D ameliorate the damaging action of inflammation on endothelial function, as vitamin D has been shown to suppress the NF-κB activity and the expression of proinflammatory cytokines (Figure 4). Nevertheless, more data are needed to clarify whether the improved endothelial function after vitamin D treatment occurs through inhibition of inflammation.

## 6. Review of Clinical Trials: The Effect of Vitamin D Supplementation on Endothelial Function

In recent decades, the efficacy of vitamin D supplementation on improving endothelial function has been investigated in several randomized controlled trials. These studies were conducted on diverse populations including healthy individuals and patients with chronic diseases such as recent history of stroke, coronary artery disease, myocardial infarction, T2D, chronic kidney disease, and peripheral artery disease [53,122,123,124,125,126,]. In addition to differences in the study populations, methodological variance exists across these studies with regard to the type and doses of vitamin D, the form of the supplementation, and the interventional duration. The most commonly used vitamin D was ergocalciferol, while a few studies used cholecalciferol and paricalcitol. These studies exclusively used oral vitamin D supplementation but utilized different forms of supplementation including capsules, tablets, solution, and fortified biscuits. Vitamin D doses ranged from 1000 IU/day to over 7000 IU/day and the duration of supplementation ranged from eight weeks at the shortest to 52 weeks at the longest [127,128,129]. Results from these studies, in conclusion, were inconsistent, and the efficacy of vitamin D in improving endothelial function appeared to be influenced by various factors such as the pathological condition of participating groups, dose of vitamin D, and the duration of supplementation. Previous findings suggested that higher doses of, or sustained vitamin D supplementation, may be effective in improving the endothelial function in patients with T2D or chronic kidney disease. Sudden et al. have shown that a single dose of 100,000 IU of ergocalciferol induced a significant increase in endothelial function, assessed via FMD, eight weeks after the administration in patients with T2D [130]. Furthermore, a single dose of 300,000 IU of cholecalciferol supplementation was effective for improving brachial artery FMD eight weeks after the administration in non-diabetic patients with chronic kidney disease [131]. Similarly, the improvement of brachial artery FMD was detected in chronic kidney disease patients after receiving a weekly dose of 50,000 IU of cholecalciferol for 12 weeks [132]. On the other hand, those beneficial effects of vitamin D administration were not detected at the lower doses in the same disease conditions [133,134]. Furthermore, a majority of the studies were conducted for 12–16 weeks and only one study supplemented the participants with vitamin D for a year. It is possible that improved endothelial function may be detected if the sufficient vitamin D concentration was maintained for a long period time.

## 7. Conclusions

In the present literature review, we aimed to provide insight into the role of vitamin D in regulating endothelial function. The previous preclinical studies have supported the biochemical links between the genomic effect of vitamin D and regulation of endothelial function, which involves regulation of the bioavailability and bioactivity of the predominant endothelium-derived vasodilator, NO. In contrast, the available evidence is insufficient to conclude the efficacy of vitamin D supplementation for improvement of endothelial function in humans, although there is some evidence of benefit with very high dose vitamin D supplementation. Evidence is lacking regarding the use of lower dose supplementation for longer durations, such as one year or longer. Therefore, future studies with longer supplement duration with a wide range of vitamin D doses are needed to determine the efficacy, optimal dosing, and optimal duration of vitamin D supplementation to improve endothelial function in humans.

## References

[1] Fleming I., Busse R. (1999). Signal transduction of eNOS activation. Cardiovasc. Res..

[2] Dedeoglu M., Garip Y., Bodur H. (2014). Osteomalacia in Crohn’s disease. Arch. Osteoporos..

[3] Kanikarla-Marie P., Jain S.K. (2016). 1,25(OH) 2 D 3 inhibits oxidative stress and monocyte adhesion by mediating the upregulation of GCLC and GSH in endothelial cells treated with acetoacetate (ketosis). J. Steroid Biochem. Mol. Biol..

[4] Bischoff H., Borchers M., Gudat F., Duermueller U., Theiler R., Stähelin H., Dick W. (2001). In Situ Detection of 1,25-dihydroxyvitamin D Receptor In human Skeletal Muscle Tissue. Histochem. J..

[5] Napoli C., De Nigris F., Williams-Ignarro S., Pignalosa O., Sica V., Ignarro L.J. (2006). Nitric oxide and atherosclerosis: An update. Nitric Oxide Biol. Chem..

[6] González-Molero I., Rojo-Martínez G., Morcillo S., Gutiérrez C., Rubio-Martín E., Pérez-Valero V., Esteva I., De Adana M.S.R., Almaraz M.C., Colomo N. (2013). Hypovitaminosis D and incidence of obesity: A prospective study. Eur. J. Clin. Nutr..

[7] Sokol S.I., Srinivas V., Crandall J.P., Kim M., Tellides G., Lebastchi A., Yu Y., Gupta A.K., Alderman M.H. (2012). The effects of vitamin D repletion on endothelial function and inflammation in patients with coronary artery disease. Vasc. Med..

[8] Labudzynskyi D.O., Zaitseva O.V., Latyshko N.V., Gud Kova O.O., Veliky M.M. (2015). Vitamin D3 contribution to the regulation of oxidative metabolism in the liver of diabetic mice. Ukr. Biochem. J..

[9] Barthelmes J., Nägele M.P., Ludovici V., Ruschitzka F., Sudano I., Flammer A.J. (2017). Endothelial dysfunction in cardiovascular disease and Flammer syndrome-similarities and differences. EPMA J..

[10] Zhang Q.-Y., Jiang C., Sun C., Tang T.-F., Jin B., Cao D.-W., He J., Zhang M. (2014). Hypovitaminosis D is associated with endothelial dysfunction in patients with non-dialysis chronic kidney disease. J. Nephrol..

[11] Equils O., Naiki Y., Shapiro A.M., Michelsen K., Lu D., Adams J., Jordan S. (2006). 1,25-Dihydroxyvitamin D 3 inhibits lipopolysaccharide-induced immune activation in human endothelial cells. Clin. Exp. Immunol..

[12] Zella J.B., DeLuca H.F. (2003). Vitamin D and autoimmune diabetes. J. Cell. Biochem..

[13] Holick M.F. (1996). Vitamin D and bone health. J. Nutr..

[14] Deluca H.F., Cantorna M.T. (2001). Vitamin D: Its role and uses in immunology. FASEB J..

[15] Dulak J., Józkowicz A., Dembinska-Kiec A., Guevara I., Zdzienicka A., Zmudzinska-Grochot D., Florek I., Wójtowicz A., Szuba A., Cooke J.P. Nitric oxide induces the synthesis of vascular endothelial growth factor by rat vascular smooth muscle cells. Arterioscler. Thromb. Vasc. Biol..

[16] Bouillon R., Carmeliet G., Verlinden L., Van Etten E., Verstuyf A., Luderer H.F., Lieben L., Mathieu C., DeMay M. (2008). Vitamin D and human health: Lessons from vitamin D receptor null mice. Endocr. Rev..

[17] Radomski M.W., Palmer R.M.J., Moncada S. (1987). The role of nitric oxide and cGMP in platelet adhesion to vascular endothelium. Biochem. Biophys. Res. Commun..

[18] Ramagopalan S.V., Heger A., Berlanga A.J., Maugeri N.J., Lincoln M.R., Burrell A., Handunnetthi L., Handel A., Disanto G., Orton S.-M. (2010). A ChIP-seq defined genome-wide map of vitamin D receptor binding: Associations with disease and evolution. Genome Res..

[19] Suzuki Y.J., Forman H.J., Sevanian A. (1997). Oxidants as stimulators of signal transduction. Free Radic. Biol. Med..

[20] Haussler M.R., Haussler C.A., Jurutka P.W., Thompson P.D., Hsieh J.C., Remus L.S., Selznick S.H., Whitfield G.K. (1997). The vitamin D hormone and its nuclear receptor: Molecular actions and disease states. J. Endocrinol..

[21] Holick M.F. (2004). Vitamin D: Importance in the prevention of cancers, type 1 diabetes, heart disease, and osteoporosis. Am. J. Clin. Nutr..

[22] Lakshmi S.V.V., Padmaja G., Kuppusamy P., Kutala V.K. (2009). Oxidative stress in cardiovascular disease. Indian J. Biochem. Biophys..

[23] Hingorani A.D. (2001). Polymorphisms in endothelial nitric oxide synthase and atherogenesis: John French Lecture 2000. Atherosclerosis.

[24] Donato A.J., Pierce G.L., Lesniewski L.A., Seals D.R. (2009). Role of NFkappaB in age-related vascular endothelial dysfunction in humans. Aging.

[25] Feghali C.A., Wright T.M. (1997). Cytokines in acute and chronic inflammation. Front. Biosci..

[26] Haussler M.R., Whitfield G.K., Kaneko I., Haussler C.A., Hsieh D., Hsieh J.C., Jurutka P.W. (2013). Molecular mechanisms of vitamin D action. Calcif. Tissue Int..

[27] Forrest K.Y.Z., Stuhldreher W.L. (2011). Prevalence and correlates of vitamin D deficiency in US adults. Nutr. Res..

[28] Dharmashankar K., Widlansky M.E. (2010). Vascular endothelial function and hypertension: Insights and directions. Curr. Hypertens. Rep..

[29] Guzik T.J., Harrison D.G. (2007). Endothelial NF-κB as a mediator of kidney damage: The missing link between systemic vascular and renal disease?. Circ. Res..

[30] Hussin A.M., Ashor A.W., Schoenmakers I., Hill T., Mathers J.C., Siervo M. (2017). Effects of vitamin D supplementation on endothelial function: A systematic review and meta-analysis of randomised clinical trials. Eur. J. Nutr..

[31] Higashi Y., Noma K., Yoshizumi M., Kihara Y. (2009). Endothelial function and oxidative stress in cardiovascular diseases. Circ. J..

[32] Sadeghi K., Wessner B., Laggner U., Ploder M., Tamandl D., Friedl J., Zügel U., Steinmeyer A., Pollak A., Roth E. (2006). Vitamin D3 down-regulates monocyte TLR expression and triggers hyporesponsiveness to pathogen-associated molecular patterns. Eur. J. Immunol..

[33] Vazquez G., de Boland A.R., Boland R. (1997). Stimulation of Ca2+Release-Activated Ca2+Channels as a Potential Mechanism Involved in Non-Genomic 1,25(OH)2-Vitamin D3-Induced Ca2+Entry in Skeletal Muscle Cells. Biochem. Biophys. Res. Commun..

[34] Cayatte A.J., Palacino J.J., Horten K., Cohen R.A. (1994). Chronic inhibition of nitric oxide production accelerates neointima formation and impairs endothelial function in hypercholesterolemic rabbits. Arterioscler. Thromb. Vasc. Biol..

[35] Oldham K.M., Bowen P.E. (1998). Oxidative stress in critical care: Is antioxidant supplementation beneficial?. J. Am. Diet. Assoc..

[36] Igari K., Kudo T., Toyofuku T., Inoue Y. (2016). Endothelial Dysfunction of Patients with Peripheral Arterial Disease Measured by Peripheral Arterial Tonometry. Int. J. Vasc. Med..

[37] Ignarro L. (1985). The Pharmacological and Physiological Role of Cyclic GMP in Vascular Smooth Muscle Relaxation. Annu. Rev. Pharmacol. Toxicol..

[38] Tohari A.M., Alhasani R.H., Biswas L., Patnaik S.R., Reilly J., Zeng Z., Shu X. (2019). Vitamin D attenuates oxidative damage and inflammation in retinal pigment epithelial cells. Antioxidants.

[39] Abdali D., Samson S.E., Grover A.K. (2015). How effective are antioxidant supplements in obesity and diabetes?. Med. Princ. Pract..

[40] Furchgott R.F., Zawadzki J.V. (1980). The obligatory role of endothelial cells in the relaxation of arterial smooth muscle by acetylcholine. Nature.

[41] Polidoro L., Properzi G., Marampon F., Gravina G.L., Festuccia C., Di Cesare E., Scarsella L., Ciccarelli C., Zani B.M., Ferri C. (2013). Vitamin D protects human endothelial cells from H2O2 oxidant injury through the Mek/Erk-sirt1 axis activation. J. Cardiovasc. Transl. Res..

[42] Nakai K., Fujii H., Kono K., Goto S., Kitazawa R., Kitazawa S., Hirata M., Shinohara M., Fukagawa M., Nishi S. (2014). Vitamin D activates the Nrf2-keap1 antioxidant pathway and ameliorates nephropathy in diabetic rats. Am. J. Hypertens..

[43] Brandenburg V.M., Vervloet M.G., Marx N. (2012). The role of vitamin D in cardiovascular disease: From present evidence to future perspectives. Atherosclerosis.

[44] Andrukhova O., Slavic S., Zeitz U., Riesen S.C., Heppelmann M.S., Ambrisko T.D., Markovic M., Kuebler W.M., Erben R.G. (2014). Vitamin D is a regulator of endothelial nitric oxide synthase and arterial stiffness in mice. Mol. Endocrinol..

[45] Li Y.C., Pirro A.E., Amling M., Delling G., Baron R., Bronson R., Demay M.B. (1997). Targeted ablation of the vitamin D receptor: An animal model of vitamin D-dependent rickets type II with alopecia. Proc. Natl. Acad. Sci. USA.

[46] Fleet J.C. (2004). Rapid, Membrane-Initiated Actions of 1,25 Dihydroxyvitamin D: What Are They and What Do They Mean?. J. Nutr..

[47] Busse R., Fleming I. (1995). Regulation and functional consequences of endothelial nitric oxide formation. Ann. Med..

[48] Schiffrin E.L. (2002). A critical review of the role of endothelial factors in the pathogenesis of hypertension. J. Cardiovasc. Pharmacol..

[49] Umans J. (1995). Nitric Oxide in the Regulation of Blood Flow and Arterial Pressure. Annu. Rev. Physiol..

[50] Vazquez G., De Boland A.R. (1996). Involvement of protein kinase C in the modulation of 1α,25-dihydroxy-vitainin D3-induced 45Ca2+ uptake in rat and chick cultured myoblasts. Biochim. Biophys. Acta Mol. Cell Res..

[51] Busse R., Mülsch A. (1990). Calcium-dependent nitric oxide synthesis in endothelial cytosol is mediated by calmodulin. FEBS Lett..

[52] Buitrago C., Pardo V.G., Boland R. (2013). Role of VDR in 1α,25-dihydroxyvitamin D3-dependent non-genomic activation of MAPKs, Src and Akt in skeletal muscle cells. J. Steroid Biochem. Mol. Biol..

[53] Molinari C., Rizzi M., Squarzanti D.F., Pittarella P., Vacca G., Renò F. (2013). 1α,25-dihydroxycholecalciferol (vitamin D3) induces NO-dependent endothelial cell proliferation and migration in a three-dimensional matrix. Cell. Physiol. Biochem..

[54] Mazidi M., Karimi E., Rezaie P., Vatanparast H. (2016). The impact of vitamin D supplement intake on vascular endothelial function; a systematic review and meta-analysis of randomized controlled trials. Food Nutr. Res..

[55] Griendling K.K., Sorescu D., Lassègue B., Ushio-Fukai M. (2000). Modulation of protein kinase activity and gene expression by reactive oxygen species and their role in vascular physiology and pathophysiology. Arterioscler. Thromb. Vasc. Biol..

[56] Michell B.J., Griffiths J.E., Mitchelhill K.I., Rodriguez-Crespo I., Tiganis T., Bozinovski S., de Montellano P.R.O., Kemp B.E., Pearson R.B. (1999). The Akt kinase signals directly to endothelial nitric oxide synthase. Curr. Biol..

[57] Pietsch E.C., Chan J.Y., Torti F.M., Torti S.V. (2003). Nrf2 mediates the induction of ferritin H in response to xenobiotics and cancer chemopreventive dithiolethiones. J. Biol. Chem..

[58] Babaei S., Teichert-Kuliszewska K., Monge J.C., Mohamed F., Bendeck M.P., Stewart D.J. (1998). Role of nitric oxide in the angiogenic response in vitro to basic fibroblast growth factor. Circ. Res..

[59] Chen C.H., Henry P.D. (1997). Atherosclerosis as a microvascular disease: Impaired angiogenesis mediated by suppressed basic fibroblast growth factor expression. Proc. Assoc. Am. Physicians.

[60] Palmer R.M.J., Ashton D.S., Moncada S. (1988). Vascular endothelial cells synthesize nitric oxide from L-arginine. Nature.

[61] Ziche M., Morbidelli L., Masini E., Amerini S., Granger H.J., Maggi C.A., Geppetti P., Ledda F. (1994). Nitric oxide mediates angiogenesis in vivo and endothelial cell growth and migration in vitro promoted by substance P. J. Clin. Investig..

[62] Mittler R. (2002). Oxidative stress, antioxidants and stress tolerance. Trends Plant Sci..

[63] Doroudi M., Schwartz Z., Boyan B.D. (2015). Membrane-mediated actions of 1,25-dihydroxy vitamin D3: A review of the roles of phospholipase A2 activating protein and Ca^2+/^calmodulin-dependent protein kinase II. J. Steroid Biochem. Mol. Biol..

[64] Lundwall K., Jacobson S.H., Jörneskog G., Spaak J. (2018). Treating endothelial dysfunction with vitamin D in chronic kidney disease: A meta-analysis. BMC Nephrol..

[65] Ziche M., Parenti A., Ledda F., DelL’Era P., Granger H.J., Maggi C.A., Presta M. (1997). Nitric oxide promotes proliferation and plasminogen activator production by coronary venular endothelium through endogenous bFGF. Circ. Res..

[66] Meier B., Radeke H.H., Selle S., Younes M., Sies H., Resch K., Habermehl G.G. (1989). Human fibroblasts release reactive oxygen species in response to interleukin-1 or tumour necrosis factor-α. Biochem. J..

[67] Hernández-Presa M., Bustos C., Ortego M., Tuñon J., Renedo G., Ruiz-Ortega M., Egido J. (1997). Angiotensin-converting enzyme inhibition prevents arterial nuclear factor-κB activation, monocyte chemoattractant protein-1 expression, and macrophage infiltration in a rabbit model of early accelerated atherosclerosis. Circulation.

[68] Maulik N., Das D.K. (2002). Redox signaling in vascular angiogenesis. Free Radic. Biol. Med..

[69] Ning C., Liu L., Lv G., Yang Y., Zhang Y., Yu R., Wang Y., Zhu J. (2015). Lipid metabolism and inflammation modulated by Vitamin D in liver of diabetic rats. Lipids Health Dis..

[70] Kwak M.K., Egner P.A., Dolan P.M., Ramos-Gomez M., Groopman J.D., Itoh K., Yamamoto M., Kensler T.W. (2001). Role of phase 2 enzyme induction in chemoprotection by dithiolethiones. Mutat. Res. Fundam. Mol. Mech. Mutagen..

[71] Grandi N.C., Breitling L.P., Brenner H. (2010). Vitamin D and cardiovascular disease: Systematic review and meta-analysis of prospective studies. Prev. Med..

[72] Martínez-Miguel P., Valdivielso J.M., Medrano-Andrés D., Román-García P., Cano-Peñalver J.L., Rodríguez-Puyol M., Rodríguez-Puyol D., López-Ongil S. (2014). The active form of vitamin D, calcitriol, induces a complex dual upregulation of endothelin and nitric oxide in cultured endothelial cells. Am. J. Physiol. Endocrinol. Metab..

[73] Suzuki Y., Ichiyama T., Ohsaki A., Hasegawa S., Shiraishi M., Furukawa S. (2009). Anti-inflammatory effect of 1α,25-dihydroxyvitamin D3 in human coronary arterial endothelial cells: Implication for the treatment of Kawasaki disease. J. Steroid Biochem. Mol. Biol..

[74] Cosentino F., Christopher Sill J., Katušić Z.S. (1994). Role of superoxide anions in the mediation of endothelium-dependent contractions. Hypertension.

[75] Kendrick J., Andrews E., You Z., Moreau K., Nowak K.L., Farmer-Bailey H., Seals D.R., Chonchol M. (2017). Cholecalciferol, calcitriol, and vascular function in CKD a randomized, double-blind trial. Clin. J. Am. Soc. Nephrol..

[76] Zalba G., José G.S., Moreno M.U., Fortuño M.A., Fortuño A., Beaumont F.J., Díez J. (2001). Oxidative stress in arterial hypertension role of NAD(P)H oxidase. Hypertension..

[77] Verma S., Anderson T.J. (2002). Fundamentals of Endothelial Function for the Clinical Cardiologist. Circulation.

[78] Beckman J.S., Koppenol W.H. (1996). Nitric oxide, superoxide, and peroxynitrite: The good, the bad, and the ugly. Am. J. Physiol. Cell Physiol..

[79] Khafaji H.A.H., Al Suwaidi J. (2007). Endothelial dysfunction in diabetes mellitus. Vasc. Heal. Risk Manag..

[80] Zou M.-H., Shi C., Cohen R.A. (2013). Oxidation of the zinc-thiolate complex and uncoupling of endothelial nitric oxide synthase by peroxynitrite. J. Clin. Investig..

[81] Hewitt N.A., O’Connor A.A., O’Shaughnessy D.V., Elder G.J. (2013). Effects of cholecalciferol on functional, biochemical, vascular, and quality of life outcomes in hemodialysis patients. Clin. J. Am. Soc. Nephrol..

[82] Codoñer-Franch P., Tavárez-Alonso S., Simó-Jordá R., Laporta-Martín P., Carratalá-Calvo A., Alonso-Iglesias E. (2012). Vitamin D status is linked to biomarkers of oxidative stress, inflammation, and endothelial activation in obese children. J. Pediatr..

[83] Victor V.M., Rocha M., Solá E., Bañuls C., Garcia-Malpartida K., Hernandez-Mijares A. (2009). Oxidative stress, endothelial dysfunction and atherosclerosis. Curr. Pharm. Des..

[84] Kim Y.C., Masutani H., Yamaguchi Y., Itoh K., Yamamoto M., Yodoi J. (2001). Hemin-induced activation of the thioredoxin gene by Nrf2: A differential regulation of the antioxidant responsive element by a switch of its binding factors. J. Biol. Chem..

[85] Kono K., Fujii H., Nakai K., Goto S., Kitazawa R., Kitazawa S., Shinohara M., Hirata M., Fukagawa M., Nishi S. (2013). Anti-oxidative effect of vitamin d analog on incipient vascular lesion in non-obese type 2 diabetic rats. Am. J. Nephrol..

[86] Pittarella P., Squarzanti D.F., Molinari C., Invernizzi M., Uberti F., Renò F. (2015). NO-dependent proliferation and migration induced by Vitamin D in HUVEC. J. Steroid Biochem. Mol. Biol..

[87] Jain S.K., Micinski D., Huning L., Kahlon G., Bass P.F., Levine S.N. (2014). Vitamin D and L-cysteine levels correlate positively with GSH and negatively with insulin resistance levels in the blood of type 2 diabetic patients. Eur. J. Clin. Nutr..

[88] Jablonski K.L., Chonchol M., Pierce G.L., Walker A.E., Seals D.R. (2011). 25-Hydroxyvitamin D deficiency is associated with inflammation-linked vascular endothelial dysfunction in middle-aged and older adults. Hypertension.

[89] Montenegro K.R., Cruzat V., Carlessi R., Newsholme P. (2019). Mechanisms of vitamin D action in skeletal muscle. Nutr. Res. Rev..

[90] Chan K., Kan Y.W. (1999). Nrf2 is essential for protection against acute pulmonary injury in mice. Proc. Natl. Acad. Sci. USA.

[91] Cho H.Y., Jedlicka A.E., Reddy S.P.M., Kensler T.W., Yamamoto M., Zhang L.Y., Kleeberger S.R. (2002). Role of NRF2 in protection against hyperoxic lung injury in mice. Am. J. Respir. Cell Mol. Biol..

[92] Ignarro L.J. (1990). Nitric oxide. A novel signal transduction mechanism for transcellular communication. Hypertension.

[93] Kerr S., Brosnan M.J., McIntyre M., Reid J.L., Dominiczak A.F., Hamilton C.A. (1999). Superoxide anion production is increased in a model of genetic hypertension: Role of the endothelium. Hypertension.

[94] Kissner R., Nauser T., Bugnon P., Lye P.G., Koppenol W.H. (1997). Formation and properties of peroxynitrite as studied by laser flash photolysis, high-pressure stopped-flow technique, pulse radiolysis. Chem. Res. Toxicol..

[95] Pierce G.L., Lesniewski L.A., Lawson B.R., Beske S.D., Seals D.R. (2009). Nuclear factor-κB activation contributes to vascular endothelial dysfunction via oxidative stress in overweight/obese middle-aged and older humans. Circulation.

[96] Tesarik J., Mendoza C. (1997). Direct non-genomic effects of follicular steroids on maturing human oocytes: Oestrogen versus androgen antagonism. Hum. Reprod. Update.

[97] Zhang H., Zhuang X.-D., Meng F.-H., Chen L., Dong X.-B., Liu G.-H., Li J.-H., Dong Q., Xu J.-D., Yang C.-T. (2016). Calcitriol prevents peripheral RSC96 Schwann neural cells from high glucose & methylglyoxal-induced injury through restoration of CBS/H 2 S expression. Neurochem. Int..

[98] Antuna-Puente B., Feve B., Fellahi S., Bastard J.-P. (2008). Adipokines: The missing link between insulin resistance and obesity. Diabetes Metab..

[99] Ertek S., Akgül E., Cicero A.F., Kütük U., Demirtaş S., Çehreli S., Erdoǧan G. (2012). 25-hydroxy vitamin D levels and endothelial vasodilator function in normotensive women. Arch. Med. Sci..

[100] Song Y., Wang L., Pittas A.G., Del Gobbo L.C., Zhang C., Manson J.E., Hu F.B. (2013). Blood 25-hydroxy vitamin D levels and incident type 2 diabetes: A meta-analysis of prospective studies. Diabetes Care.

[101] Brezinski E., Follansbee M., Armstrong E., Armstrong A. (2014). Endothelial Dysfunction and the Effects of TNF Inhibitors on the Endothelium in Psoriasis and Psoriatic Arthritis: A Systematic Review. Curr. Pharm. Des..

[102] Bergholm R., Leirisalo-Repo M., Vehkavaara S., Mäkimattila S., Taskinen M.R., Yki-Järvinen H. (2002). Impaired responsiveness to NO in newly diagnosed patients with rheumatoid arthritis. Arterioscler. Thromb. Vasc. Biol..

[103] Ishii T., Itoh K., Takahashi S., Sato H., Yanagawa T., Katoh Y., Bannai S., Yamamoto M. (2000). Transcription factor Nrf2 coordinately regulates a group of oxidative stress-inducible genes in macrophages. J. Biol. Chem..

[104] Csiszar A., Wang M., Lakatta E.G., Ungvari Z. (2008). Inflammation and endothelial dysfunction during aging: Role of NF-κB. J. Appl. Physiol..

[105] De Winther M.P.J., Kanters E., Kraal G., Hofker M.H. (2005). Nuclear factor κB signaling in atherogenesis. Arterioscler. Thromb. Vasc. Biol..

[106] Papapetropoulos A., García-Cardeña G., Madri J.A., Sessa W.C. (1997). Nitric oxide production contributes to the angiogenic properties of vascular endothelial growth factor in human endothelial cells. J. Clin. Investig..

[107] Donato A.J., Black A.D., Jablonski K.L., Gano L.B., Seals D.R. (2008). Aging is associated with greater nuclear NFκB, reduced IκBα, and increased expression of proinflammatory cytokines in vascular endothelial cells of healthy humans. Aging Cell.

[108] Grundmann M., Haidar M., Placzko S., Niendorf R., Darashchonak N., Hubel C.A., Von Versen-Höynck F. (2012). Vitamin D improves the angiogenic properties of endothelial progenitor cells. Am. J. Physiol. Cell Physiol..

[109] Wang G.P., Deng Z.D., Ni J., Qu Z.L. (1997). Oxidized low density lipoprotein and very low density lipoprotein enhance expression of monocyte chemoattractant protein-1 in rabbit peritoneal exudate macrophages. Atherosclerosis.

[110] Zhang C., Hein T.W., Wang W., Ren Y., Shipley R.D., Kuo L. (2006). Activation of JNK and xanthine oxidase by TNF-α impairs nitric oxide-mediated dilation of coronary arterioles. J. Mol. Cell. Cardiol..

[111] Yang J., Park Y., Zhang H., Gao X., Wilson E., Zimmer W., Abbott L., Zhang C. (2009). Role of MCP-1 in tumor necrosis factor-α-induced endothelial dysfunction in type 2 diabetic mice. Am. J. Physiol. Hear. Circ. Physiol..

[112] Wang E.W., Siu P., Pang M.Y.C., Woo J., Collins A.R., Benzie I. (2017). Vitamin D deficiency, oxidative stress and antioxidant status: Only weak association seen in the absence of advanced age, obesity or pre-existing disease. Br. J. Nutr..

[113] Giulietti A., Van Etten E., Overbergh L., Stoffels K., Bouillon R., Mathieu C. (2007). Monocytes from type 2 diabetic patients have a pro-inflammatory profile. 1,25-Dihydroxyvitamin D3 works as anti-inflammatory. Diabetes Res. Clin. Pract..

[114] Holick M.F. (2007). Vitamin D deficiency. N. Engl. J. Med..

[115] Zhang Y., Leung N.Y.M., Richers B.N., Liu Y., Remigio L.K., Riches D.W., Goleva E. (2012). Vitamin D inhibits monocyte/macrophage proinflammatory cytokine production by targeting MAPK phosphatase-1. J. Immunol..

[116] Ebihara K., Masuhiro Y., Kitamoto T., Suzawa M., Uematsu Y., Yoshizawa T., Ono T., Harada H., Matsuda K., Hasegawa T. (1996). Intron retention generates a novel isoform of the murine vitamin D receptor that acts in a dominant negative way on the vitamin D signaling pathway. Mol. Cell. Biol..

[117] Sugden J.A., Davies J.I., Witham M.D., Morris A.D., Struthers A.D. (2008). Vitamin D improves endothelial function in patients with Type 2 diabetes mellitus and low vitamin D levels. Diabet. Med..

[118] Cohen-Lahav M., Shany S., Tobvin D., Chaimovitz C., Douvdevani A. (2006). Vitamin D decreases NFkappaB activity by increasing IkappaBalpha levels. Nephrol. Dial. Transpl..

[119] Revelli A., Massobrio M., Tesarik J. (1998). Nongenomic actions of steroid hormones in reproductive tissues. Endocr. Rev..

[120] Gepner A.D., Ramamurthy R., Krueger D.C., Korcarz C.E., Binkley N., Stein J.H. (2012). A prospective randomized controlled trial of the effects of Vitamin D supplementation on cardiovascular disease risk. PLoS ONE.

[121] Ni W., Watts S.W., Ng M., Chen S., Glenn D.J., Gardner D.G. (2014). Elimination of vitamin D receptor in vascular endothelial cells alters vascular function. Hypertension.

[122] Breslavsky A., Frand J., Matas Z., Boaz M., Barnea Z., Shargorodsky M. (2013). Effect of high doses of vitamin D on arterial properties, adiponectin, leptin and glucose homeostasis in type 2 diabetic patients. Clin. Nutr..

[123] Garg A., Grundy S.M., Unger R.H. (1992). Comparison of effects of high and low carbohydrate diets on plasma lipoproteins and insulin sensitivity in patients with mild NIDDM. Diabetes.

[124] Schwartz Z., Shaked D., Hardin R.R., Gruwell S., Dean D.D., Sylvia V.L., Boyan B.D. (2003). 1α,25(OH)2D3 causes a rapid increase in phosphatidylinositol-specific PLC-β activity via phospholipase A2-dependent production of lysophospholipid. Steroids.

[125] Steyers C.M., Miller F.J. (2014). Endothelial dysfunction in chronic inflammatory diseases. Int. J. Mol. Sci..

[126] Witham M.D., Dove F.J., Khan F., Lang C.C., Belch J.J.F., Struthers A.D. (2013). Effects of Vitamin D supplementation on markers of vascular function after myocardial infarction—A randomised controlled trial. Int. J. Cardiol..

[127] Hossein-Nezhad A., Mirzaei K., Keshavarz S.A., Ansar H., Saboori S., Tootee A. (2013). Evidences of dual role of vitamin D through cellular energy homeostasis and inflammation pathway in risk of cancer in obese subjects. Minerva Med..

[128] Lerman A., Burnett J.C. (1992). Intact and altered endothelium in regulation of vasomotion. Circulation.

[129] Matsunaga T., Weihrauch D.W., Moniz M.C., Tessmer J., Warltier D.C., Chilian W.M. (2002). Angiostatin inhibits coronary angiogenesis during impaired production of nitric oxide. Circulation.

[130] Stricker H., Tosi Bianda F., Guidicelli-Nicolosi S., Limoni C., Colucci G. (2012). Effect of a single, oral, high-dose vitamin D supplementation on endothelial function in patients with peripheral arterial disease: A randomised controlled pilot study. Eur. J. Vasc. Endovasc. Surg..

[131] Chitalia N., Ismail T., Tooth L., Boa F., Hampson G., Goldsmith D., Kaski J.C., Banerjee D. (2014). Impact of vitamin D supplementation on arterial vasomotion, stiffness and endothelial biomarkers in chronic kidney disease patients. PLoS ONE.

[132] Zhang Q., Zhang M., Wang H., Sun C., Feng Y., Zhu W., Cao D., Shao Q., Li N., Xia Y. (2018). Vitamin D supplementation improves endothelial dysfunction in patients with non-dialysis chronic kidney disease. Int. Urol. Nephrol..

[133] Kato S. The function of vitamin D receptor in vitamin D action. J. Biochem..

[134] Yiu Y.F., Yiu K.H., Siu C.W., Chan Y.H., Li S.W., Wong L.Y., Lee S.W.L., Tam S., Wong E.W.K., Lau C.P. (2013). Randomized controlled trial of vitamin D supplement on endothelial function in patients with type 2 diabetes. Atherosclerosis.

